# The efficacy and safety of radiotherapy and immunotherapy with or without anlotinib in driver gene-negative advanced non-small cell lung cancer

**DOI:** 10.3389/fonc.2026.1694207

**Published:** 2026-02-09

**Authors:** Weijian Miao, Jingjing Sun, QiMeng Tao, Yan Zhou, Hao Jiang

**Affiliations:** 1 Department of Radiation Oncology, The First Affiliated Hospital of Bengbu Medical University, Bengbu, Anhui, China; 2 Joint Research Center for Regional Diseases of Institute of Health and Medicine (IHM), Bengbu Medical University, Bengbu, Anhui, China; 3 Joint Research Center for Regional Diseases of Institute of Health and Medicine (IHM), The First Affiliated Hospital of Bengbu Medical University, Bengbu, Anhui, China; 4 Anhui Provincial Key Laboratory of Tumor Evolution and Intelligent Diagnosis and Treatment, Bengbu Medical University, Bengbu, Anhui, China

**Keywords:** anlotinib, efficacy, immune checkpoint inhibitors, non-small cell lung cancer (NSCLC), radiotherapy, safety

## Abstract

**Background:**

Radiotherapy paired with immune checkpoint inhibitors (ICIs) has benefited patients with driver gene-negative NSCLC, although treatment resistance remains an obstacle. Anlotinib, a broad-spectrum tyrosine kinase inhibitor, has potential to reinforce immunotherapy by modifying the tumor environment. This study investigates whether incorporating anlotinib can amplify the efficacy of combined treatment protocols.

**Methods:**

A total of 203 individuals diagnosed with stage IIIB-IV NSCLC were retrospectively assessed. Treatment occurred at the First Affiliated Hospital of Bengbu Medical University between 2021 and 2023. Patients were grouped into those receiving radiotherapy and immunotherapy (n=123) and those receiving anlotinib in addition (n=80). Clinical outcomes assessed included PFS, OS, response metrics, and treatment-related side effects.

**Results:**

Over a median follow-up of 26 months, the group receiving anlotinib showed enhanced median PFS (10.0 months vs 6.0 months, P = 0.043 HR: 0.708, 95% CI: 0.496–1.009). However, there was no statistically significant difference in overall survival (OS) between the two groups (median OS: 20.0 vs. 18.0 months; P = 0.344, HR:0.848 95% CI:0.597-1.205). the RT+IO+A regimen demonstrated a 10% higher ORR than the RT+IO regimen (45.0% vs. 35.0%), while the DCR was similar between the two groups (88.8% vs. 91.1%). Toxic effects were manageable but more frequent in the triple-therapy group.

**Conclusion:**

The triple regimen improved disease stabilization but did not yield OS benefits. Due to the increased toxicity associated with the addition of anlotinib, Future research is required to weigh advantages against added toxicity.

## Background

Lung cancer remains the most lethal malignancy worldwide, accounting for an estimated 1.8 million deaths and over 2.4 million new cases annually, as reported by GLOBOCAN in its Global Cancer Statistics 2022 (1). Non-small cell lung cancer (NSCLC) constitutes approximately 85% of all lung cancer cases and is often diagnosed at advanced stages, where curative treatments are limited and prognosis remains poor (2, 3). In China alone, more than 1.06 million new lung cancer cases and 733,300 deaths were reported in 2022, underscoring a substantial and ongoing disease burden (4). Despite advances in therapy, the 5-year survival rate for advanced NSCLC remains below 20%, highlighting the urgent need for more effective treatment strategies (5).

In recent years, targeted therapies against driver gene mutations such as EGFR, ALK, and ROS1 have significantly improved outcomes for patients with driver-positive NSCLC (6, 7). Emerging evidence also underscores the potential of combining TKIs with immunotherapy. For instance, anti-angiogenic TKIs can normalize tumor vasculature and alleviate immunosuppression, thereby enhancing ICI efficacy (8, 9), Clinical trials exploring such combinations, including those evaluating PD-(L)1 inhibitors with anti-angiogenic TKIs, have shown promising efficacy in NSCLC, particularly as a strategy to overcome resistance (10, 11). However, treatment remains challenging for those with driver-negative tumors. Immune checkpoint inhibitors (ICIs) targeting programmed cell death protein 1 (PD-1), its ligand PD-L1, and cytotoxic T-lymphocyte–associated antigen 4 (CTLA-4) have reshaped the treatment landscape for advanced NSCLC. Pivotal clinical trials, including KEYNOTE-024, CheckMate-227, and IMpower110, have demonstrated that ICIs improve both progression-free survival (PFS) and overall survival (OS) in patients with high PD-L1 expression and other predictive biomarkers (12–14). Nevertheless, primary or acquired resistance to ICIs limits long-term responses in a considerable proportion of patients, particularly those with low tumor mutational burden or an immunosuppressive tumor microenvironment (15–17). This has prompted the investigation of synergistic combination therapies to overcome immune resistance.

Radiotherapy (RT), traditionally utilized for local tumor control, has emerged as a potential immune-modulating partner for ICIs. Preclinical studies indicate that RT can upregulate PD-L1 expression on tumor cells, thereby augmenting the efficacy of PD-1/PD-L1 blockade (14). Clinical validation of this synergy was provided by the PACIFIC trial, in which durvalumab consolidation after chemoradiotherapy significantly improved survival in patients with unresectable stage III NSCLC (18). Subsequent trials such as GEMSTONE-301 and LAURA are further exploring this strategy across different molecular subgroups and disease stages (19, 20).

Despite these promising results, challenges including adaptive resistance, local recurrence, and metastatic progression persist. Anti-angiogenic agents represent a complementary therapeutic approach by promoting tumor vascular normalization, alleviating hypoxia, and enhancing immune cell infiltration (21). Anlotinib, a multi-target tyrosine kinase inhibitor that blocks VEGFR, FGFR, PDGFR, and c-Kit signaling, has demonstrated both anti-proliferative and immune-modulating effects in advanced NSCLC (22, 23). Mechanistically, it remodels the tumor microenvironment to facilitate drug penetration and reverse immune exclusion.

The combination of anlotinib and docetaxel has been shown to significantly improve PFS and objective response rate (ORR) compared with docetaxel monotherapy in patients with advanced NSCLC who progressed after platinum-based chemotherapy (24). Preliminary clinical evidence also suggests that anlotinib may enhance ICI efficacy and prolong disease control, particularly in pretreated or ICI-resistant populations (25–27). These findings support the notion that anlotinib can potentiate the effects of both cytotoxic and immune-targeting therapies. Therefore, combining anti-angiogenic agents with immunomodulatory functions together with immunotherapy and radiotherapy—known for eliciting abscopal effects—may yield deeper and more synergistic antitumor responses, offering a new therapeutic option for this patient population.

This retrospective study aims to evaluate the efficacy and safety of combining radiotherapy, immune checkpoint inhibition, and anlotinib in patients with advanced NSCLC. By assessing outcomes such as PFS, OS, ORR, disease control rate (DCR), and treatment-related adverse events, we seek to generate clinically relevant insights that may inform the design of future prospective trials and help optimize combination strategies for this challenging cohort.

### Patients

This retrospective study included 203 patients with stage IIIB-IV NSCLC who received lung lesion radiotherapy at the First Affiliated Hospital of Bengbu Medical University from January 2021 to December 2023. Patients were divided into two groups: radiotherapy + immunotherapy + anlotinib (RT+IO+A, n=80) and radiotherapy + immunotherapy (RT+IO, n=123). The inclusion criteria were as follows: (1) Age ≥18 years; (2) Histologically or cytologically confirmed advanced (IIIB-IV) NSCLC; (3) Eastern Cooperative Oncology Group (ECOG) performance status (PS) score of 0-2; (4) Measurable disease according to RECIST 1.1 standards. (5) Driver gene negative: This is defined as the absence of driver gene mutations. It includes cases with sensitive mutations (such as EGFR, ALK, ROS1) or cases with unknown mutations but showing effective responses to targeted therapy. The exclusion criteria were as follows: (1) Contraindications to radiotherapy; (2) Presence of other malignancies; (3) Significant organ dysfunction; (4) Allergies to study drugs; (5) Uncontrolled infections or autoimmune diseases.

### Treatment details and data collection

Radiotherapy was delivered per institutional standards for advanced NSCLC, with intent for radical or palliative treatment as clinically indicated. The specific prescription dose, fractionation scheme, and target volumes (primary lung tumor and/or metastatic sites) were determined by the treating radiation oncologist based on disease stage, lesion location, and performance status. Immunotherapy consisted of PD-1 inhibitors administered intravenously every 3 weeks. Anlotinib was administered orally at a starting dose of 12mg once daily (2 weeks on/1 week off). The drug dose was adjusted according to the adverse reactions of patients, Treatment duration was continued until disease progression, unacceptable toxicity, or patient withdrawal.

The timing of radiotherapy relative to the initiation of immunotherapy and anlotinib was not protocol-defined and varied in clinical practice. Radiotherapy could be delivered concurrently with (overlapping cycles) or sequentially to (before or after) systemic therapy, based on multidisciplinary team decisions and patient-specific factors such as symptom burden and disease pace.

Due to the retrospective, real-world nature of this study, detailed parameters including exact radiotherapy doses, fractionation, delineated target volumes, and the precise start dates relative to systemic therapy cycles were not uniformly captured in a structured format for all 203 patients. Data collection focused on treatment modality exposure (RT, IO, A) and key clinical outcomes. This limitation in granular treatment data is acknowledged below.

### Endpoints

The patient will undergo initial follow-up 4–8 weeks after radiation therapy, followed by evaluation every 3–6 months. We will systematically collect comprehensive clinical data for all participants, including gender, age, smoking history, histological subtypes, TNM staging, immunotherapy cycles, and immunotherapy drugs. Tumor response was assessed every 3 cycles of treatment using contrast-enhanced computed tomography (CT) scans in accordance with the Response Evaluation Criteria in Solid Tumors (RECIST) version 1.1, which defines therapeutic outcomes as follows: complete response (CR), indicating the disappearance of all target lesions and reduction of any pathological lymph nodes in short axis to <10 mm; partial response (PR), representing at least a 30% decrease in the sum of diameters of target lesions; stable disease (SD), denoting neither sufficient shrinkage to qualify as PR nor sufficient increase to qualify as progressive disease; and progressive disease (PD), characterized by at least a 20% increase in the sum of diameters of target lesions, an absolute increase of at least 5 mm, and/or the appearance of new lesions. All radiographic images were independently reviewed by two experienced radiologists who were unaware of the patients’ treatment assignments or clinical outcomes. Any discrepancies in assessment were resolved through consensus or by adjudication from a third senior radiologist. The primary endpoint is progression free survival (PFS), defined as the time interval from the start of radiation therapy to disease progression, death, or the last follow-up. Secondary endpoints include: overall survival (OS; time from radiotherapy to death or last follow-up); Objective response rate (ORR; proportion of CR/PR achieved); Disease control rate (DCR; proportion of CR/PR/SD achieved); And adverse events (according to CTCAE v5.0 grading), including blood toxicity (neutropenia, thrombocytopenia, anemia, leukopenia), gastrointestinal events (nausea/vomiting), fatigue, hypertension, radiation pneumonitis/esophagitis, and immune related adverse events (irAE).

### Statistical analysis

Statistical analyses were performed using R software, Continuous variables that follow a normal distribution are described using mean and standard deviation, while those that do not follow a normal distribution are described using median and interquartile range. Categorical variables are described using frequency. We used chi-square test and Fisher’s exact test to compare ORR and adverse reactions between two groups. Kaplan-Meier method was used to evaluate PFS and OS, and Cox proportional hazards model was used for univariate and multivariate analysis. P < 0.05 was considered statistically significant. In multivariate Cox regression analyses, adjustments were made for clinically relevant covariates, including age, presence of brain metastases, ECOG performance status, disease stage, and other metastatic sites (liver, bone, etc.), to control for potential confounding effects arising from baseline imbalances between treatment groups. Given the variability and incomplete granularity of radiotherapy parameters and treatment sequencing data, these factors were not included as covariates in the primary multivariate Cox regression model. The analysis focused on comparing the two treatment strategy groups (RT+IO vs. RT+IO+A) as defined by their exposure to the respective modalities.

## Results

### Patient characteristics

This study enrolled 203 patients with advanced non-small cell lung cancer (NSCLC), stratified into RT+IO+A (radiotherapy + immunotherapy + anlotinib, n=80) and RT+IO (radiotherapy + immunotherapy, n=123). The overall cohort was predominantly male (156, 76.9%), with a median age of 64.08 ± 9.69 years, predominantly stage IVA/IVB disease (150, 73.9%), and squamous cell carcinoma histology (119, 58.6%); lung metastases were the most frequent site (98, 48.3%), and 14.8% of patients had ≥3 metastatic organs. Intergroup comparisons revealed RT+IO+A patients were significantly younger (62.08 ± 10.18 years vs 65.38 ± 9.16 years) with a higher proportion of non-smokers (45.0% vs 34.2%), and lower brain metastasis incidence (18.8% vs 25.2%), while RT+IO had a higher proportion of PD-L1 high expression (≥50%) (9.8% vs 3.8%),. Primary immunotherapy agents comprised tislelizumab (88, 43.3%), sintilimab (64, 31.5%), and camrelizumab (39, 19.2%); camrelizumab usage was significantly higher in RT+IO+A (25.0% vs 15.4%), whereas sintilimab was more prevalent in RT+IO (35.8% vs 25.0%), with secondary agents including cadonilimab (6 cases). The proportion receiving ≥4 immunotherapy cycles was comparable between groups (78.8% vs 77.2%). Importantly, the groups were well-balanced regarding gender distribution (male: 76.3% vs 77.2%), histology (squamous: 53.8% vs 61.8%), T stage (T3-4: 55.0% vs 65.0%), N stage (N2-3: 92.5% vs 93.5%), M stage (M1: 73.8% vs 74.8%), liver metastasis (7.5% vs 8.1%), bone metastasis (17.5% vs 15.4%), and ECOG performance status (0-1: 88.8% vs 88.6%) (**Table 1**).

**Table 1:** The baseline characteristics of patients.

Variables	Total (n = 203)	RT+IO (n = 123)	RT+IO+A (n = 80)
AGE, Mean ± SD	64.08 ± 9.69	65.38 ± 9.16	62.08 ± 10.18
SEX, n(%)			
Female	47 (23.15)	28 (22.76)	19 (23.75)
Male	156 (76.85)	95 (77.24)	61 (76.25)
T, n(%)			
1	12 (5.91)	9 (7.32)	3 (3.75)
2	67 (33.00)	34 (27.64)	33 (41.25)
3	74 (36.45)	50 (40.65)	24 (30.00)
4	50 (24.63)	30 (24.39)	20 (25.00)
N, n(%)			
1	14 (6.90)	8 (6.50)	6 (7.50)
2	116 (57.14)	69 (56.10)	47 (58.75)
3	73 (35.96)	46 (37.40)	27 (33.75)
M, n(%)			
0	52 (25.62)	31 (25.20)	21 (26.25)
1	151 (74.38)	92 (74.80)	59 (73.75)
Disease stage, n(%)			
IIIB, IIIC	52 (25.62)	31 (25.20)	21 (26.25)
IVA, IVB	151 (74.38)	92 (74.80)	59 (73.75)
Lung metastases, n(%)			
NO	105 (51.72)	63 (51.22)	42 (52.50)
YES	98 (48.28)	60 (48.78)	38 (47.50)
Brain metastases, n(%)			
NO	157 (77.34)	92 (74.80)	65 (81.25)
YES	46 (22.66)	31 (25.20)	15 (18.75)
Liver metastases, n(%)			
NO	187 (92.12)	113 (91.87)	74 (92.50)
YES	16 (7.88)	10 (8.13)	6 (7.50)
Bone metastases, n(%)			
NO	170 (83.74)	104 (84.55)	66 (82.50)
YES	33 (16.26)	19 (15.45)	14 (17.50)
Others, n(%)			
NO	158 (77.83)	95 (77.24)	63 (78.75)
YES	45 (22.17)	28 (22.76)	17 (21.25)
Number of transferred organs, n(%)			
<3	182 (89.66)	108 (87.80)	74 (92.50)
≥3	21 (10.34)	15 (12.20)	6 (7.50)
Smoking, n(%)			
Never	78 (38.42)	42 (34.15)	36 (45.00)
Current or former	125 (61.58)	81 (65.85)	44 (55.00)
ECOG, n(%)			
0	14 (6.90)	6 (4.88)	8 (10.00)
1	166 (81.77)	103 (83.74)	63 (78.75)
2	23 (11.33)	14 (11.38)	9 (11.25)
Histologic characteristic, n(%)			
Adenocarcinoma	84 (41.38)	47 (38.21)	37 (46.25)
Squamous cell carcinoma	119 (58.62)	76 (61.79)	43 (53.75)
Chemotherapy, n(%)			
Concurrent	69 (33.99)	42 (34.15)	27 (33.75)
sequential	134 (66.01)	81 (65.85)	53 (66.25)
PDL1 tumor proportion score, n(%)			
Unknown	180 (88.67)	109 (88.62)	71 (88.75)
<1	4 (1.97)	2 (1.63)	2 (2.50)
1-49	4 (1.97)	0 (0.00)	4 (5.00)
≥50	15 (7.39)	12 (9.76)	3 (3.75)
Immunotherapy cycle, n(%)			
<4	45 (22.17)	28 (22.76)	17 (21.25)
≥4	158 (77.83)	95 (77.24)	63 (78.75)
Immunotherapy drugs, n(%)			
Trelizumab	88 (43.35)	52 (42.28)	36 (45.00)
Carilizumab	39 (19.21)	19 (15.45)	20 (25.00)
Sintilimab Injection	64 (31.53)	44 (35.77)	20 (25.00)
Cadonilimab	6 (2.96)	2 (1.63)	4 (5.00)
Others	6 (2.96)	6 (4.88)	0 (0.00)

RT, radiotherapy; IO, immunotherapy; A, anlotinib; ECOG, Eastern Cooperative Oncology Group Performance Status.

### Follow-up and survival outcomes

**Figure 1:**
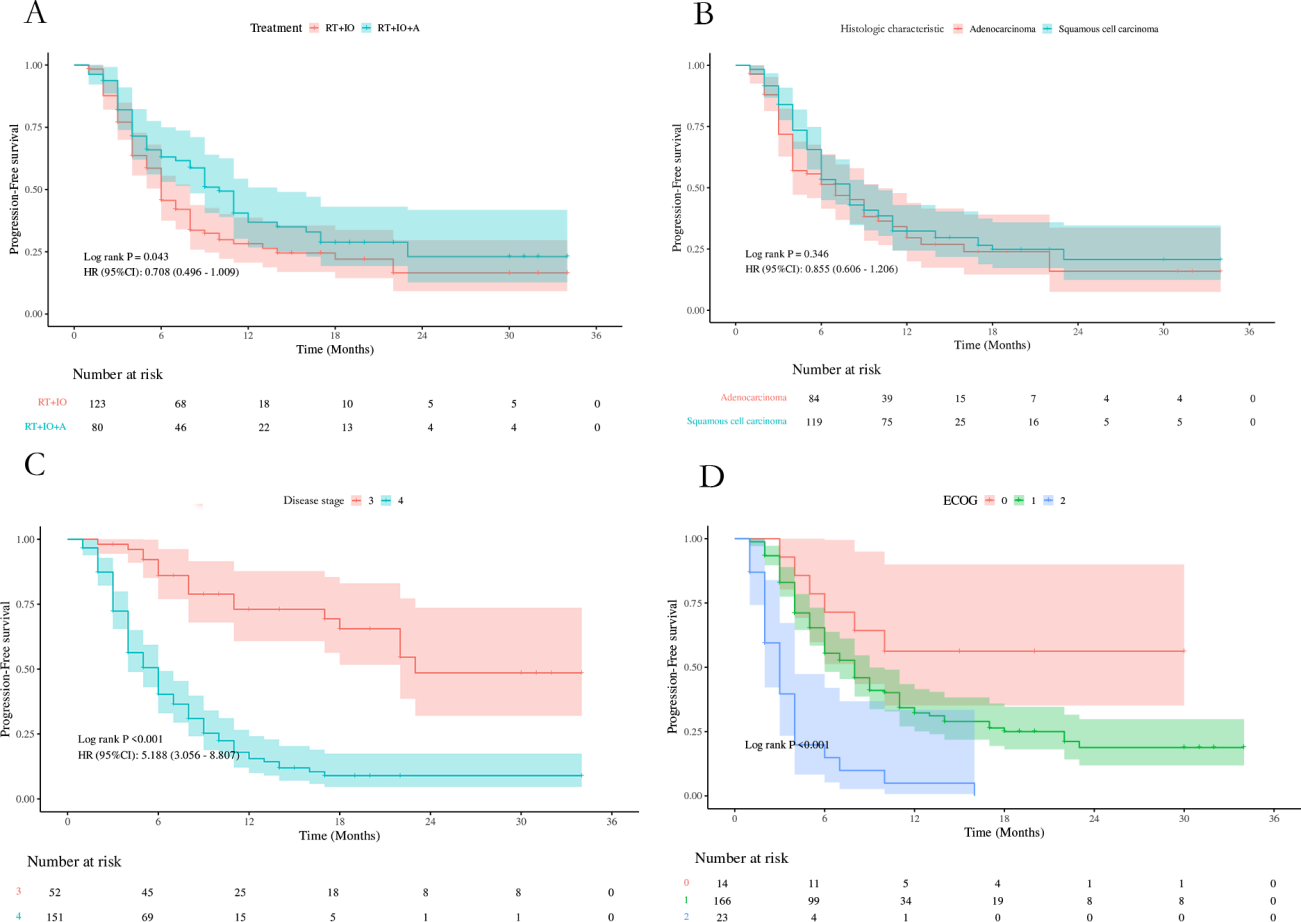
**(A–D)** Depict the clinical parameters that affect Progression-Free survival (PFS) in univariate analysis [**(A)** treatment; **(B)** Histologic characteristic; **(C)** Installment; **(D)** Eastern Cooperative Oncology Group Performance Status (ECOG)].

**Figure 2:**
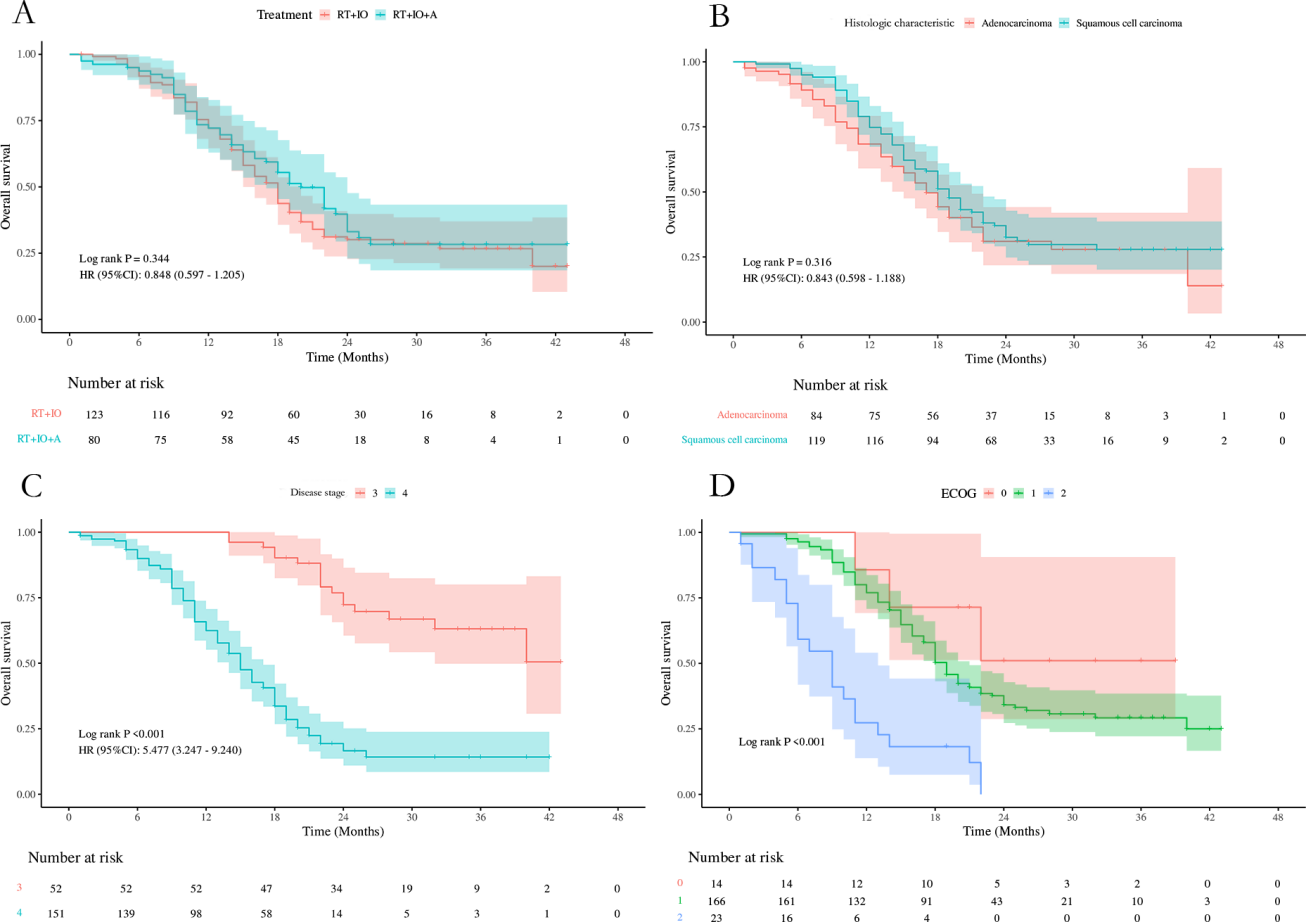
**(A–D)** depict the clinical parameters that affect OS in univariate analysis [**(A)** Treatment; **(B)** Histologic characteristic; **(C)** Disease stage; **(D)** Eastern Cooperative Oncology Group Performance Status (ECOG)].

Patients were followed up periodically until the study cutoff date (May 31, 2025). The follow-up duration was calculated from the date of initial treatment to the date of the last known contact, death, or the study cutoff date. The median follow-up time was 26.0 months (range: 5.0–53.0 months). In the group receiving radiotherapy combined with anlotinib and immunotherapy (RT+IO+A), the median progression-free survival (PFS) was 10.0 months (95% CI: 8.00–14.00), and the median overall survival (OS) was 20.0 months (95% CI: 17.00–24.00). In the group receiving only radiotherapy combined with immunotherapy (RT+IO), the median PFS was 6.0 months (95% CI: 6.00–8.00), and the median OS was 18.0 months (95% CI: 15.00–20.00). A statistically significant difference was observed in PFS between the two groups (P = 0.043, HR: 0.708, 95% CI: 0.496–1.009), but no significant difference was observed in OS (P = 0.344, HR:0.848 95% CI:0.597-1.205)(**Figures 1**, **2**).

### Objective response rate and disease control rate

In RT+IO+A (radiotherapy + immunotherapy + anlotinib), the ORR was 45.0%, and the DCR was 88.8%. In RT+IO (radiotherapy + immunotherapy), the ORR was 35.0%, and the DCR was 91.1%. RT+IO+A demonstrated a 10% higher ORR than RT+IO (45.0% vs. 35.0%). This result is consistent with the mechanism of antiangiogenic drug (anlotinib) enhancing tumor vascular normalization and enhancing immune therapy response. It is worth noting that RT+IO had a higher disease control rate (DCR = 91.1%) and a slightly higher disease control rate (DCR = 88.8%) compared to RT+IO+A, as more patients were in a stable state of disease (SD = 56.1%) due to the lack of concomitant use of antiangiogenic drugs, which may be related to the delayed effect of immunotherapy.

### Subgroup analysis

**Figure 3:**
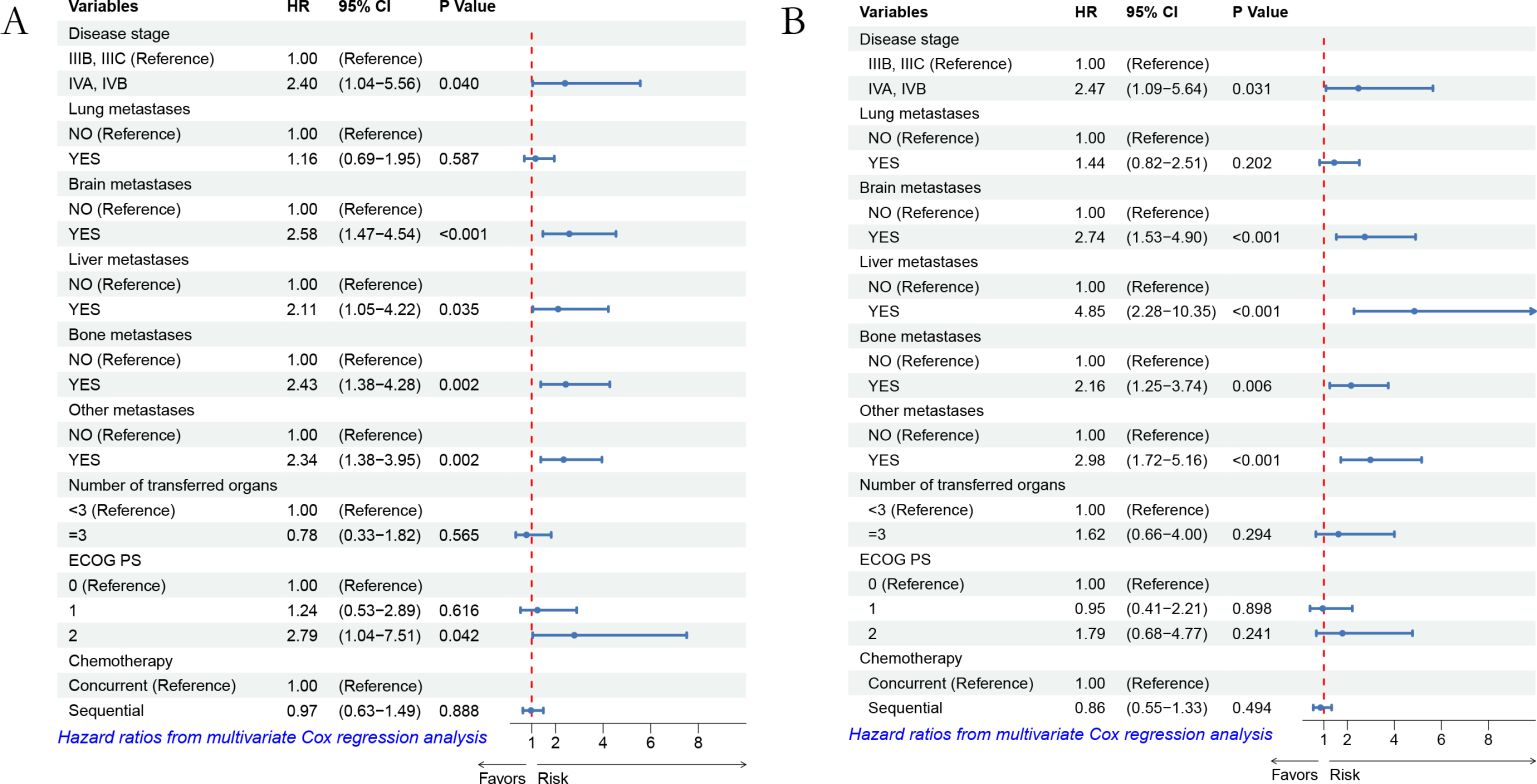
**(A)** is the forest plot of PFS multi factor analysis, **(B)** is the forest plot of OS multi factor analysis.

Using Cox regression analysis to explore potential factors affecting the prognosis of combination therapy. Univariate analysis showed that stage IV disease, lung, liver, brain, bone or other organ metastasis, synchronous radiotherapy and chemotherapy, ≥ 3 metastatic organs, and ECOG performance status were significantly correlated with PFS and OS. Variables with P-values<0.05 in univariate analysis were included in multivariate Cox regression. Multivariate analysis showed that the ECOG scores of liver, brain, bone, and other organ metastases were significantly correlated with PFS, while the ECOG scores of liver, brain, bone, and other organ metastases were significantly correlated with OS. ECOG-PS 0–1 patients showed better PFS under combination therapy. In addition, patients without distant metastases such as brain, liver, bone, or other organs have a better prognosis. (**Tables 2**, **3**, **Figure 3**).

**Table 2:** Single factor and multi factor Cox regression analysis of progression-free survival.

Variables	β	S.E	Z	P	HR (95%CI)
Treatment					
RT+IO					1.00 (Reference)
RT+IO+A	-0.35	0.18	-1.91	0.056	0.71 (0.50 ~ 1.01)
SEX					
Female					1.00 (Reference)
Male	0.15	0.21	0.74	0.457	1.17 (0.78 ~ 1.74)
T					
1					1.00 (Reference)
2	0.22	0.38	0.57	0.570	1.24 (0.59 ~ 2.62)
3	-0.38	0.39	-0.99	0.323	0.68 (0.32 ~ 1.46)
4	0.20	0.39	0.52	0.605	1.23 (0.57 ~ 2.64)
N					
1					1.00 (Reference)
2	-0.52	0.33	-1.58	0.114	0.60 (0.31 ~ 1.13)
3	-0.09	0.33	-0.26	0.794	0.92 (0.48 ~ 1.76)
Disease stage					
IIIB, IIIC					1.00 (Reference)
IVA, IVB	1.65	0.27	6.10	<.001	5.19 (3.06 ~ 8.81)
Lung metastases					
NO					1.00 (Reference)
YES	0.50	0.17	2.87	0.004	1.65 (1.17 ~ 2.32)
Brain metastases					
NO					1.00 (Reference)
YES	1.15	0.20	5.88	<.001	3.16 (2.15 ~ 4.64)
Liver metastases					
NO					1.00 (Reference)
YES	0.84	0.27	3.13	0.002	2.32 (1.37 ~ 3.92)
Bone metastases					
NO					1.00 (Reference)
YES	0.66	0.22	2.95	0.003	1.93 (1.25 ~ 2.98)
Others					
NO					1.00 (Reference)
YES	1.12	0.20	5.72	<.001	3.06 (2.09 ~ 4.50)
Number of transferred organs					
<3					1.00 (Reference)
≥3	1.45	0.26	5.61	<.001	4.25 (2.56 ~ 7.05)
Smoking					
Never					1.00 (Reference)
Current or former	-0.03	0.18	-0.15	0.877	0.97 (0.69 ~ 1.38)
ECOG					
0					1.00 (Reference)
1	0.70	0.42	1.68	0.093	2.02 (0.89 ~ 4.61)
2	1.96	0.47	4.20	<.001	7.12 (2.85 ~ 17.80)
Histologic characteristic					
Adenocarcinoma					1.00 (Reference)
Squamous cell carcinoma	-0.16	0.18	-0.89	0.371	0.85 (0.61 ~ 1.21)
PDL1 tumor proportion score					
Unknown					1.00 (Reference)
<1	0.47	0.59	0.81	0.418	1.61 (0.51 ~ 5.06)
1-49	-0.77	0.71	-1.07	0.283	0.46 (0.11 ~ 1.88)
≥50	-0.33	0.39	-0.85	0.393	0.72 (0.33 ~ 1.54)
Chemotherapy					
Concurrent					1.00 (Reference)
sequential	0.57	0.19	2.95	0.003	1.77 (1.21 ~ 2.59)
Immunotherapy cycle					
<4					1.00 (Reference)
≥4	-0.15	0.20	-0.72	0.469	0.86 (0.58 ~ 1.29)
Immunotherapy drugs					
Trelizumab					1.00 (Reference)
Carilizumab	-0.15	0.23	-0.66	0.510	0.86 (0.54 ~ 1.35)
Sintilimab Injection	-0.24	0.21	-1.19	0.234	0.78 (0.52 ~ 1.17)
Cadonilimab	0.44	0.47	0.95	0.344	1.55 (0.62 ~ 3.87)
Others	-0.64	0.59	-1.09	0.278	0.53 (0.17 ~ 1.68)
Variables	β	S.E	Z	P	HR (95%CI)
Disease stage					
IIIB, IIIC					1.00 (Reference)
IVA, IVB	0.88	0.43	2.05	0.040	2.40 (1.04 ~ 5.56)
Lung metastases					
NO					1.00 (Reference)
YES	0.15	0.27	0.54	0.587	1.16 (0.69 ~ 1.95)
Brain metastases					
NO					1.00 (Reference)
YES	0.95	0.29	3.29	0.001	2.58 (1.47 ~ 4.54)
Liver metastases					
NO					1.00 (Reference)
YES	0.75	0.35	2.11	0.035	2.11 (1.05 ~ 4.22)
Bone metastases					
NO					1.00 (Reference)
YES	0.89	0.29	3.07	0.002	2.43 (1.38 ~ 4.28)
Others					
NO					1.00 (Reference)
YES	0.85	0.27	3.17	0.002	2.34 (1.38 ~ 3.95)
Number of transferred organs					
<3					1.00 (Reference)
≥3	-0.25	0.43	-0.58	0.565	0.78 (0.33 ~ 1.82)
ECOG					
0					1.00 (Reference)
1	0.22	0.43	0.50	0.616	1.24 (0.53 ~ 2.89)
2	1.03	0.51	2.03	0.042	2.79 (1.04 ~ 7.51)
Chemotherapy					
Concurrent					1.00 (Reference)
sequential	-0.03	0.22	-0.14	0.888	0.97 (0.63 ~ 1.49)

RT, radiotherapy; IO, immunotherapy; A, anlotinib; ECOG, Eastern Cooperative Oncology Group Performance Status.

Single factor and multi factor Cox regression analysis of progression-free survival.

**Table 3:** Single factor and multi factor Cox regression analysis of overall survival.

Variables	β	S.E	Z	P	HR (95%CI)
Treatment					
RT+IO					1.00 (Reference)
RT+IO+A	-0.16	0.18	-0.92	0.357	0.85 (0.60 ~ 1.20)
SEX					
Female					1.00 (Reference)
Male	0.08	0.20	0.39	0.696	1.08 (0.73 ~ 1.62)
T					
1					1.00 (Reference)
2	0.12	0.38	0.31	0.758	1.12 (0.53 ~ 2.37)
3	-0.57	0.39	-1.47	0.141	0.57 (0.26 ~ 1.21)
4	0.03	0.39	0.07	0.947	1.03 (0.48 ~ 2.21)
N					
1					1.00 (Reference)
2	-0.51	0.33	-1.56	0.119	0.60 (0.32 ~ 1.14)
3	-0.11	0.33	-0.32	0.748	0.90 (0.47 ~ 1.72)
Disease stage					
IIIB, IIIC					1.00 (Reference)
IVA, IVB	1.70	0.27	6.37	<.001	5.48 (3.25 ~ 9.24)
Lung metastases					
NO					1.00 (Reference)
YES	0.55	0.17	3.15	0.002	1.73 (1.23 ~ 2.43)
Brain metastases					
NO					1.00 (Reference)
YES	1.32	0.20	6.66	<.001	3.76 (2.55 ~ 5.55)
Liver metastases					
NO					1.00 (Reference)
YES	1.72	0.29	5.97	<.001	5.61 (3.19 ~ 9.88)
Bone metastases					
NO					1.00 (Reference)
YES	0.61	0.22	2.74	0.006	1.83 (1.19 ~ 2.83)
Others					
NO					1.00 (Reference)
YES	1.38	0.20	6.96	<.001	3.99 (2.70 ~ 5.89)
Number of transferred organs					
<3					1.00 (Reference)
≥3	2.53	0.30	8.48	<.001	12.53 (6.99 ~ 22.48)
Smoking					
Never					1.00 (Reference)
Current or former	-0.06	0.18	-0.35	0.727	0.94 (0.66 ~ 1.33)
ECOG					
0					1.00 (Reference)
1	0.62	0.42	1.49	0.137	1.87 (0.82 ~ 4.25)
2	1.94	0.47	4.16	<.001	6.96 (2.79 ~ 17.36)
Histologic characteristic					
Adenocarcinoma					1.00 (Reference)
Squamous cell carcinoma	-0.17	0.18	-0.98	0.329	0.84 (0.60 ~ 1.19)
PD-L1 tumor proportion score					
Unknown					1.00 (Reference)
<1	0.35	0.58	0.60	0.547	1.42 (0.45 ~ 4.47)
1-49	-0.57	0.71	-0.80	0.423	0.56 (0.14 ~ 2.29)
≥50	-0.54	0.39	-1.39	0.164	0.58 (0.27 ~ 1.25)
Chemotherapy					
Concurrent					1.00 (Reference)
Sequential	0.66	0.20	3.33	<.001	1.93 (1.31 ~ 2.83)
Immunotherapy cycle					
<4					1.00 (Reference)
≥4	-0.07	0.20	-0.35	0.730	0.93 (0.62 ~ 1.39)
Immunotherapy drugs					
Trelizumab					1.00 (Reference)
Carilizumab	-0.24	0.23	-1.03	0.304	0.79 (0.50 ~ 1.24)
Sintilimab Injection	-0.30	0.21	-1.48	0.139	0.74 (0.49 ~ 1.10)
Cadonilimab	0.20	0.47	0.43	0.667	1.22 (0.49 ~ 3.04)
Others	-0.69	0.59	-1.16	0.246	0.50 (0.16 ~ 1.61)
Variables	β	S.E	Z	P	HR (95%CI)
Disease stage					
IIIB, IIIC					1.00 (Reference)
IVA, IVB	0.91	0.42	2.15	0.031	2.47 (1.09 ~ 5.64)
Lung metastases					
NO					1.00 (Reference)
YES	0.36	0.28	1.27	0.202	1.44 (0.82 ~ 2.51)
Brain metastases					
NO					1.00 (Reference)
YES	1.01	0.30	3.40	<0.001	2.74 (1.53 ~ 4.90)
Liver metastases					
NO					1.00 (Reference)
YES	1.58	0.39	4.09	<0.001	4.85 (2.28 ~ 10.35)
Bone metastases					
NO					1.00 (Reference)
YES	0.77	0.28	2.76	0.006	2.16 (1.25 ~ 3.74)
Others					
NO					1.00 (Reference)
YES	1.09	0.28	3.90	<0.001	2.98 (1.72 ~ 5.16)
Number of transferred organs					
<3					1.00 (Reference)
≥3	0.48	0.46	1.05	0.294	1.62 (0.66 ~ 4.00)
ECOG					
0					1.00 (Reference)
1	-0.06	0.43	-0.13	0.898	0.95 (0.41 ~ 2.21)
2	0.58	0.50	1.17	0.241	1.79 (0.68 ~ 4.77)
Chemotherapy					
Concurrent					1.00 (Reference)
Sequential	-0.15	0.22	-0.68	0.494	0.86 (0.55 ~ 1.33)

RT, radiotherapy; IO, immunotherapy; A, anlotinib; ECOG, Eastern Cooperative Oncology Group Performance Status.

Single factor and multi factor Cox regression analysis of overall survival.

### Safety profile

In terms of security analysis. In RT+IO+A (radiotherapy+immunotherapy+anlotinib, n=80), fatigue was the most common event (grade 1-2: 56 cases [70.0%]; grade ≥3: 8 cases [10.0%]). Hematological toxicity includes leukopenia (grade 1-2: 32 cases [40.0%]; grade ≥3: 6 cases [7.5%]), thrombocytopenia (grade 1-2: 24 cases [30.0%];grade ≥3: 4 cases [5.0%]), and anemia (grade 1-2: 24 cases [30.00%] grade ≥3: 4 cases [5.0%]). Anlotinib related manifestations include hypertension (grade 1-2: 20 cases [25.0%]; grade ≥3: 6 cases [7.5%]), hand foot skin reactions (grade1-2: 16 cases [20.0%];grade ≥3: 4 cases [5.0%]), and proteinuria (grade1-2: 12 cases [15.0%];grade ≥3–2 cases [2.5%]). Gastrointestinal events include nausea/vomiting (grade 1-2: 20 cases [25.0%]; grade ≥3: 3 cases [3.8%]), diarrhea (grade1-2: 12 cases [15.0%];grade ≥3–2 cases [2.5%]), and anorexia (16 cases [20.0%]). (grade1-2: 16 cases 23.8%; grade ≥3: 3 [3.8%]) were recorded with radiation/immune pneumonia (undifferentiated etiology). Other observational results include rash/itching (22 cases [27.5%]), elevated transaminase levels (18 cases [22.5%]), thyroid dysfunction (13 cases [16.3%]), bleeding (10 cases [12.5%]), and dyslipidemia (13 cases [16.3%]). In RT+IO (radiotherapy+immunotherapy, n=123), the incidence of fatigue was 90 cases (73.1%; grade 1-2: 80 cases [65.0%]; grade ≥3:10 cases [8.1%]). Hematological events included leukopenia (grade 1-2: 43 cases [35.0%]; grade ≥3: 6 cases [4.9%]), thrombocytopenia (grade 1-2:31 cases [25.2%];grade ≥3:4 cases [3.3%]), and anemia (grade1-2: 31 cases [25.2%];grade≥3:4 cases [3.3%]). There were 8 cases of hypertension (6.5%; grade ≥3: 1cases 0.8%), with no grade ≥ 3 hand foot reactions or proteinuria. 26 cases of pneumonia (21.1%; grade ≥ 3:4 cases [3.3%]). Gastrointestinal toxicity includes nausea/vomiting (grade 1-2: 25 cases [20.3%]; grade ≥3: 4 cases [3.3%]), diarrhea (grade1-2:12 cases [9.8%]; grade≥ 3:1cases [0.8%]), and anorexia (grade1-2:18 cases [14.6%]; grade ≥3: 2 cases [1.6%]). Other events include rash/itching (27 cases [22.0%]), elevated transaminase levels (19 cases [15.4%]), thyroid dysfunction (16 cases [13.0%]), and dyslipidemia (13 cases [10.6%]). In both cohorts, ≤ 10% of patients experienced liver dysfunction and elevated bilirubin levels. The main adverse events were below grade 3, and all adverse reactions were controlled through symptomatic treatment. There were no treatment-related deaths (**Table 4**).

**Table 4:** Adverse events.

Adverse event	Group RT+IO+A (n=80)		Group RT+IO (n=123)	
	Any grade n (%)	≥3 Grade n (%)	Any grade n (%)	≥3 Grade n (%)
Fatigue	64 (80.0)	8 (10.0)	90 (73.1)	10 (8.1)
Leukopenia	38 (47.5)	6 (7.5)	49 (39.9)	6 (4.9)
Thrombocytopenia	28 (35.0)	4 (5.0)	35 (28.5)	4 (3.3)
Erythrocytopenia	23 (28.8)	3 (3.8)	27 (21.9)	2 (1.6)
Radiation/Immune Pneumonitis	19 (23.8)	3 (3.8)	26 (21.2)	4 (3.3)
Rash/Pruritus	22 (27.5)	2 (2.5)	27 (21.9)	2 (1.6)
Thyroid Dysfunction	13(16.3)	1 (1.3)	16 (13.0)	1 (0.8)
ALT/AST Increase	18 (22.5)	2 (2.5)	19 (15.4)	1 (0.8)
Bilirubin Increase	9 (11.3)	1 (1.3)	11 (8.9)	1 (0.8)
Anemia	28 (35.0)	4 (5.0)	35 (28.5)	4 (3.3)
Hypertension	26 (32.5)	6 (7.5)	7 (5.7)	1 (0.8)
Hand-Foot Skin Reaction	20 (25.0)	4 (5.0)	2 (1.6)	0 (0.0)
Proteinuria	14 (17.5)	2 (2.5)	4 (3.3)	0 (0.0)
Bleeding	10 (12.5)	2 (2.5)	3 (2.4)	1 (0.8)
Dyslipidemia	13 (16.3)	1 (1.3)	13 (10.6)	1 (0.8)
Anorexia	18 (22.5)	2 (2.5)	20 (16.2)	2 (1.6)
Nausea/Vomiting	23 (28.8)	3 (3.8)	29 (23.6)	4 (3.3)
Diarrhea	14 (17.5)	2 (2.5)	13 (10.6)	1 (0.8)
Hepatic Dysfunction	9 (11.3)	1 (1.3)	11 (8.9)	1 (0.8)

Adverse reactions in RT+IO+A and RT+IO groups.

## Discussion

The management of advanced non-small cell lung cancer (NSCLC) is increasingly shifting toward multimodal strategies. Combining radiotherapy (RT), immune checkpoint inhibitors (ICIs), and antiangiogenic agents is anticipated to overcome drug resistance and improve clinical outcomes. Anlotinib, a multi-target tyrosine kinase inhibitor, acts on several key protumorigenic pathways, including VEGFR, PDGFR, and FGFR. Its capacity to modulate the tumor microenvironment via vascular normalization and enhanced immune infiltration provides a rationale for combining it with RT and ICIs. In this study, anlotinib was added to radiotherapy and immunotherapy (RT+IO+A) to exploit this potential synergy.

Growing evidence supports this concept. In patients with locally advanced NSCLC, the combination of anlotinib and RT resulted in a higher objective response rate (ORR) and reduced tumor marker levels compared with RT alone. The potential of anlotinib to reverse immune resistance and prolong ICI efficacy has also been observed in prior studies, where the combination therapy group exhibited improved disease control rate (DCR) and progression-free survival (PFS). However, intensifying treatment must be balanced against the associated toxicity burden. Anlotinib commonly leads to hypertension and hand-foot syndrome, although these adverse events are predominantly grade 1–2 and can generally be managed with supportive care. Previous clinical trials, including those in glioblastoma, have demonstrated that adding anlotinib to standard regimens does not significantly increase the incidence of grade ≥3 toxicities or raise unexpected safety concerns (28). These findings support the feasibility of the triple-combination strategy in clinical practice.

In this study, we observed that the addition of anlotinib to radiotherapy and immunotherapy significantly prolonged progression-free survival (PFS) compared with radiotherapy and immunotherapy alone. However, this PFS benefit did not translate into a statistically significant improvement in overall survival (OS). The discrepancy between PFS and OS outcomes may be attributed to several factors. First, the impact of subsequent therapies is a major consideration. As a retrospective analysis, crossover or administration of other effective treatments (such as subsequent antiangiogenic agents, including anlotinib) in the control group after disease progression may mitigate the initial survival advantage of the experimental group, thereby obscuring potential OS differences. Although our data did not systematically capture detailed post-progression treatments, clinical records suggest that a notable proportion of RT+IO patients later received anlotinib, which may have contributed to the lack of OS difference observed. Second, inherent limitations of our study design—including its retrospective nature and limited sample size—may have resulted in insufficient statistical power to detect a moderate yet clinically meaningful OS benefit. Although no OS difference was observed, the marked improvement in PFS underscores the enhanced disease control and clinical value of incorporating anlotinib into the combination regimen. This may be particularly relevant in palliative care settings and warrants further validation in prospective randomized controlled trials. It is also important to acknowledge certain baseline imbalances between the two groups, as presented in the patient characteristics table. Specifically, the RT+IO+A cohort was younger and had a lower prevalence of brain metastases. Such imbalances are inherent to retrospective, non-randomized studies and may reflect therapeutic selection bias in clinical practice—for instance, a tendency to avoid intensive triplet regimens in patients with active brain metastases due to safety concerns or perceived limited intracranial efficacy. Importantly, these variables were rigorously adjusted for in multivariate analyses, in which neither age nor brain metastasis status significantly influenced the treatment effect on PFS. Moreover, the consistent direction and magnitude of PFS benefit across key subgroups support the robustness of the primary finding. Nonetheless, these observations highlight the need for prospective randomized trials to eliminate selection bias and confirm these results.

Additionally, we noted an interesting finding: the triple-therapy group did not demonstrate superior OS compared to the dual-therapy group, and in some analyses, OS was even numerically shorter. This paradox may be explained by baseline imbalances due to nonrandom treatment allocation. Specifically, patients with stage III disease, who generally have a lower tumor burden and more favorable prognosis, were more frequently assigned to the RT+ICI group, possibly to avoid overtreatment. In contrast, those with stage IV disease, characterized by extensive metastases and poorer prognosis, were more likely to receive triple therapy (RT+IO+A). This selection bias may have led to an overrepresentation of high-risk patients in the RT+IO+A group, thereby skewing the OS comparison. Previous studies have consistently shown that the median OS for stage III NSCLC can exceed 40 months with ICI consolidation after chemoradiotherapy, whereas stage IV disease remains largely incurable, typically exhibiting a median OS of less than 21 months even with aggressive combination therapies (29).

Patients with stage IV disease, due to higher tumor burden and greater heterogeneity, are generally more prone to therapeutic resistance and exhibit poorer tolerance to intensified treatment regimens. However, the observed disparity in overall survival outcomes in this study may be influenced by baseline imbalances resulting from nonrandom treatment allocation. Although the overall stage distribution was similar between groups, the RT+IO+A cohort included a higher proportion of patients with high-risk features, such as multiple metastatic sites and involvement of specific organs (e.g., liver, bone). These factors have been independently associated with inferior survival, as confirmed in our multivariate analysis. Clinicians may have been more inclined to select triple therapy for patients with more aggressive disease, inadvertently introducing selection bias that could obscure potential survival benefits from the treatment itself. Consequently, these baseline imbalances likely attenuated the survival benefit associated with the addition of anlotinib in this cohort, explaining the absence of a significant overall survival improvement despite better disease control metrics. Therefore, interpretation of the between-group differences in overall survival should be made with caution, acknowledging the impact of pretreatment characteristic disparities (30, 31).

For patients with stage III disease, the treatment objective is often curative. Hence, the use of intensified therapy such as triple combination should be approached with caution. Although anlotinib has shown efficacy in extending OS as a monotherapy in advanced NSCLC, its integration with RT and ICI in this setting raises concerns about cumulative toxicity, particularly immune-related adverse events (irAEs) and radiation pneumonitis. In potentially curable patients, these risks may compromise long-term outcomes by necessitating treatment interruptions or discontinuation.

In contrast, for stage IV patients whose treatment goal is prolongation of life and disease control, the addition of anlotinib offers mechanistic and clinical advantages. By inhibiting VEGFR2, anlotinib reduces tumor interstitial pressure, enhances oxygenation, and improves RT sensitivity. Concurrently, its inhibition of PDGFR-β and FGFR1 facilitates vascular normalization and promotes T-cell infiltration, thereby enhancing ICI efficacy (26, 32, 33). Clinical studies in small-cell lung cancer with brain metastases have confirmed that combining anlotinib with stereotactic RT can significantly extend intracranial PFS, supporting its role in aggressive disease scenarios (34).

An additional observation in our cohort is the off-label, early-line use of anlotinib in some patients with stage IV disease. Although anlotinib is conventionally reserved for third-line settings, its earlier integration is gaining traction, especially in patients with high tumor burden or specific molecular characteristics. Mechanistically, early use may potentiate treatment efficacy by modifying the tumor microenvironment before resistance mechanisms are fully established. Recent clinical trials lend credence to this strategy (35). The CAMPASS Phase III study demonstrated that in PD-L1–positive patients, anlotinib plus an anti-PD-L1 antibody significantly prolonged PFS versus ICI monotherapy, with more pronounced benefits in those with PD-L1 ≥50% expression. Similarly, the ALTER-L001 trial showed that combining anlotinib with EGFR-TKIs after slow progression yielded substantial PFS gains (36). These findings underscore the potential of early anlotinib intervention in specific patient subsets.

Nonetheless, the number of patients receiving first- or second-line anlotinib in our study was limited, precluding statistically powered Subgroup Analysis. The retrospective design and non-standardized treatment criteria further introduce selection bias (37). Therefore, while our findings suggest that early anlotinib use may be beneficial, its efficacy and safety profile must be validated through prospective randomized controlled trials. In summary, this study demonstrates that radiotherapy combined with immunotherapy and anlotinib improves PFS in advanced NSCLC, particularly in patients with high disease burden. Although the toxicity profile observed was consistent with expectations for this combination regimen—and therefore considered manageable under appropriate clinical supervision—it underscores the necessity of careful and continuous monitoring. The absence of OS improvement further highlights the importance of prudent patient selection and proactive toxicity management. The promising role of early anlotinib intervention warrants further investigation in well-designed clinical trials. Future research should also focus on identifying predictive biomarkers to guide therapeutic strategies and optimize individualized treatment.

This study has several limitations. First, its retrospective, single-center design may introduce selection bias. Crucially, detailed radiotherapy parameters (including dose, fractionation, and target volumes) and the precise timing of radiotherapy relative to the initiation and cycles of immunotherapy and anlotinib were not systematically standardized or uniformly documented for all patients. This heterogeneity reflects real-world practice but limits the reproducibility of our specific regimen and the ability to analyze the impact of treatment sequencing on outcomes. Second, incomplete medical records precluded accurate assessment of PD-L1 expression in a substantial subset. Second, incomplete medical records precluded accurate assessment of PD-L1 expression in a substantial subset of patients, limiting a precise evaluation of its predictive role in this combination therapy. Third, despite multivariate adjustments, residual confounding cannot be fully excluded due to the non-randomized treatment allocation. Notably, patients in the RT+IO+A group were younger and had fewer brain metastases, which might have affected outcomes independently of treatment. Although these factors were statistically controlled, unmeasured variables—such as physician preference, patient adherence, or undocumented comorbidities—could further contribute to bias. Prospective clinical trials or multicenter real-world studies are needed to validate these findings and to better define the optimal treatment model and dosing strategy for immune checkpoint inhibitors combined with anti-angiogenic agents in advanced driver gene-negative NSCLC. Future studies should aim to provide higher-level evidence to guide clinical practice in this setting.

## Conclusion

This study suggests that the addition of anlotinib to radiotherapy combined with immunotherapy (triplet therapy) significantly prolonged median progression-free survival (PFS) in patients with advanced EGFR wild-type non-small cell lung cancer. However, no significant improvement was observed in overall survival (OS). The incorporation of anlotinib was associated with a marked increase in targeted therapy-related toxicities, Importantly, triplet therapy did not significantly elevate the risk of radiation pneumonitis or immune-related adverse events. These results imply that while triplet therapy may delay disease progression, its toxicity profile warrants careful consideration, and patient selection should be performed cautiously. Further prospective, biomarker-driven studies are warranted to validate these findings and explore potential survival benefits.

## Data Availability

The raw data supporting the conclusions of this article will be made available by the authors, without undue reservation.
